# Testicular “Hyperstimulation” Syndrome: A Case of Functional Gonadotropinoma

**DOI:** 10.1155/2014/194716

**Published:** 2014-01-28

**Authors:** Astha Thakkar, Subramanian Kannan, Amir Hamrahian, Richard A. Prayson, Robert J. Weil, Charles Faiman

**Affiliations:** ^1^Topiwala National Medical College and BYL Nair Charitable Hospital, Mumbai 400008, India; ^2^Endocrinology, Diabetes & Metabolism Institute, Cleveland Clinic Foundation, Cleveland, OH 44195, USA; ^3^Institute of Anatomic Pathology, Cleveland Clinic Foundation, Cleveland, OH 44195, USA; ^4^Brain Tumor & Neuro-Oncology Center, Department of Neurosurgery, The Neurological Institute, Cleveland Clinic Foundation, Cleveland, OH 44195, USA

## Abstract

Gonadotropins secreting pituitary tumors tend to present as sellar mass with hypogonadism. Biologically active LH secretion by these tumors resulting in elevated testosterone is extremely rare. We report a case of a 48-year-old male patient who presented with giant pituitary tumor, elevated testosterone, and elevated levels of gonadotropins. Surgical resection of the tumor resulted in normalization of gonadotropins and fall in serum testosterone to subnormal levels in the postoperative period confirming that the tumor was secreting bioactive luteinizing hormone (LH).

## 1. Introduction

Most of the clinically nonfunctioning pituitary tumors are gonadotropin secreting tumors [1]. The initial manifestations of these gonadotroph tumors are visual defects, headache, and associated symptoms of anterior pituitary hormone deficiency [1]. Gonadotroph pituitary adenomas are inefficient producers and secretors of gonadotroph hormones: luteinizing hormone (LH), follicle-stimulating hormone (FSH), and the *α*-subunit of pituitary glycoprotein hormones [1]. Biologically active LH secretion by these tumors resulting in elevated testosterone is extremely rare. We report a case of a 48-year-old male patient who presented with giant pituitary tumor, elevated testosterone, and elevated levels of gonadotropins.

## 2. Case Summary

**Figure 1:**
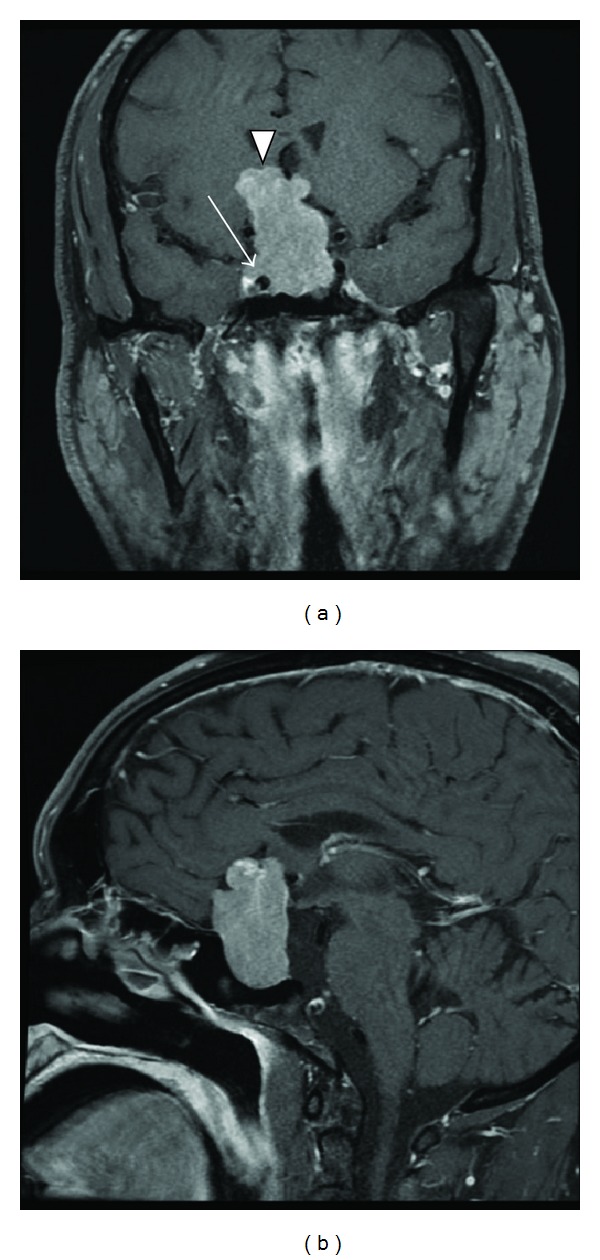
MRI of the sella. T1-weighted, postgadolinium coronal (a) and sagittal (b) images show a hyperdense lesion that expands the sella and extends intracranially (arrow head) through the suprasellar space to compress the optic nerves and chiasm. In the coronal view, one can see the tumor wrapping above the right carotid artery in the cavernous sinus (arrow).

**Figure 2:**
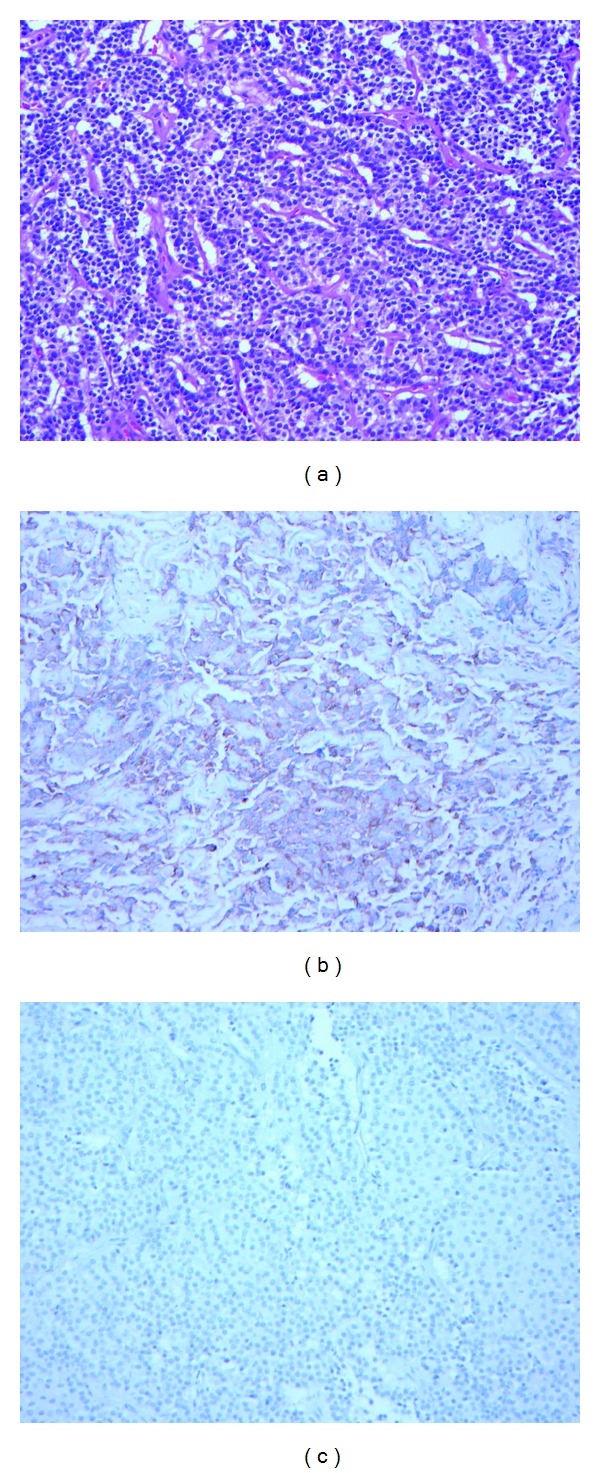
(a) H&E staining (magnification ×200) is characterized by many chromophobic epithelioid cells arranged in “pseudorosette” patterns. (b) Positive immunostaining of the tumor for FSH antibody and M3504 polyclonal antibody (DAKO, Carpinteria, CA) in 1 : 200 dilution. (c) Negative immunostaining for LH antibody (in (c)), M3502, and C93 clone (DAKO) in 1 : 320 dilution.

A 48-year-old man was referred for further management of a large pituitary tumor. His wife reported his having an increased libido for the preceding 12 months. Visual examination showed a mydriatic right pupil with a diminished light response and a right temporal field defect. There was no gynecomastia. Genital examination revealed testicular volumes approximating 25–30 cc. Biochemical evaluation showed an elevated total testosterone level of 1647 ng/dL (normal 220–1000; chemiluminescence immunoassay) and free testosterone of 515.1 pg/mL (normal 40–240; chemiluminescence immunoassay and ultrafiltration), with corresponding follicular-stimulating hormone (FSH) and LH levels of 32.7 mU/mL (normal 1–10) and 11.5 mU/mL (normal 1–7), respectively, consistent with a functional gonadotropinoma. There was no other hormonal cosecretion (Table 1). Magnetic resonance imaging (MRI) of the sella revealed a 4.5 × 3.3 × 2.5 cm sellar and suprasellar tumor, with optic nerve and chiasm compression and right cavernous sinus invasion (Figure 1). The patient underwent transsphenoidal surgery with dramatic reductions in postoperative total and free testosterone levels to 128 and 18.5, along with normalization of FSH and LH levels (6.0 mU/mL and 1.5 mU/mL), respectively. Postoperative MRI showed significant tumor debulking. Immunohistochemically, the adenoma (Figure 2(a)) stained positively for FSH (Figure 2(b)) but did not stain for LH (Figure 2(c)). Staining was repeated on different specimens of the tumor and the tumor consistently did not stain for LH. Slides prepared were not conducive for electron microscopy.

## 3. Discussion

Most gonadotropinomas are nonfunctional and clinically present as a pituitary macroadenoma and hypopituitarism [1, 2]. Immunostaining of these tumors is variable for FSH and/or LH, often unevenly distributed [3], and with the intensity of staining not correlated with plasma levels of gonadotropins [4]. There are multiple cases reported in the literature in which a functional FSH-secreting tumor results in spontaneous ovarian hyperstimulation syndrome [5–9] and of testicular enlargement [10–12]. Functional LH secreted by the tumor is extremely unusual and to our knowledge our case is the only fifth reported case in the literature [13–16]. The fact that the tumor did not immunostain using a specific monoclonal antibody against human LH, despite markedly elevated serum levels, suggests that the secreted LH was structurally different from human LH, with different epitopes detected only by the serum assay. Nonetheless, as evident from the elevated testosterone levels falling to low levels postoperatively, LH bioactivity was clearly retained. In vitro molecular analysis and electron microscopy of the tumor were not feasible with the samples available. Ultrasound of the scrotum to detect macroorchidism and serum inhibin levels was ordered to confirm the functionality of the FSH secreted; however, these results were not available at the time of publication.

## Figures and Tables

**Table 1:** Preoperative and postoperative laboratory blood tests in the patient.

Test (normal range)	Preoperatively	Postoperatively
IGF-1 (52–328 ng/mL)	64	—
Free thyroxine index (6–11 mcg/dL)	3.8	—
ACTH (8–42 pg/mL)	18	17
Basal cortisol	9.8	20.4
Postsynacthen cortisol (normal > 18 mcg/dL)	20.1	—
Prolactin (2–14 ng/mL)	14	—
FSH (1–10 mIU/mL)	32.7	6
LH (1–7 mIU/mL)	11.5	1.5
Testosterone (total) (220–1000 ng/dL)	1647	128
Testosterone (free) (40–240 pg/mL)	515.1	18.5
17-*β* estradiol (<200 pg/mL)	344	173
